# Annotations

**Published:** 1895-11-09

**Authors:** 


### Annotations.

### Medical Men and Medical Women.

The fact that both the Royal colleges have within a fortnight been asked to grant their diplomas to women has served to bring again into prominence the one-time exciting question of the fitness of medical practice as a career for women. Doubtless it may be said that this question has long ago been decided, and that the matter really before the Royal College of Physicians was as to their admission to its own diplomas. But it is to be noted that the speakers who wished to exclude women from the Royal colleges fell back to a large extent upon the arguments which were employed twenty years ago against their admission to any diplomas at all. Nevertheless, a new point was made, and a new stone was cast at woman, for it was gravely asked what use women had made of the opportunities which they now for so many years had possessed. "What contributions to medicine," said one speaker, "had proceeded from the female practitioners in America during the fifty years or so of their work there, or from those in England? Had they done anything to raise the standard of medical ethics, and how would medical questions be assisted in debate or otherwise by their partaking in them?" Now, is this quite fair? What opportunities, what facilities, and what encouragement have medical women received from their medical brethren to enter with them into the serious work of medical science as distinct from medical practice? None. It is notorious that they are excluded from the medical societies, and that for many years they were not admitted even into the British Medical Association. No helping hand, and no offer of assistance or companionship in their new work, has been offered by medical men to medical women, and we hold that it is unfair now to turn round and twit them with their barrenness of any "contributions" to medical science.

### A Gleam of Hope for the Cancer-stricken.

The conviction is gaining ground that cancer is a parasitic, that is, a microbial, disease, like tuberculosis. If this should prove to be so, the cure of several large classes of cancer cases is within sight. The results of operations during the past fifteen years certainly point in this direction. Speaking at the British Medical Association's annual congress, in July last, Mr. Jonathan Hutchinson made the assertion that cancer statistics would have to be rewritten, so large had been the proportion of cures during the last decade or two. But there are certain qualifications in this otherwise satisfactory progress. The most important of these are that the cancers which have been "cured" on so considerable a scale have been on or near the external surface of the body; and they have been diagnosed and operated upon at very early stages of their growth. Cancers of internal organs, which are seldom diagnosed very early, and which cannot be reached by the surgeon's knife, still present the same hopeless features as before. On the point of early diagnosis and operation, Dr. Roux, of Lausanne, has collected some important statistics. According to these, certain classes of cancer which offer reasonable hopes of cure if operated upon early, are lost in as many as 62 per cent. of cases for the simple reason that they are brought to the operator a few months too late. In another class, still more favourable, 12 per cent. seek operation when all hope is past, and as many as 50 per cent. present themselves when it is too late to do anything but palliative operations. The moral for all persons is that in every case where the least suspicion of the presence of a new growth is entertained, medical advice should be sought without the loss of an hour; and the urgent warning to family practitioners is that so soon as they are convinced of the presence of a new growth they should take the operating surgeon into their councils without the delay of a day.

### The Parents' Point of View.

The flood of neurotic literature from which we are just emerging was extremely interesting to the mental pathologist when at its height, and into his hands falls the wreckage as the waters subside. A thoughtful man could foresee that some case similar to that which has attracted so much attention at Battersea was bound to arise. While we consider her misguided and blind to the true interests of her sex, we would do full justice to the young lady's high character. No one can impute any but the purest motives for her action. Her convictions must be strong indeed when she sacrifices father, mother, and all her relations for them. It is for the parents that we are chiefly concerned. All our sympathies are with them. We hold that, not only were they perfectly justified in what they did, but we believe they would have neglected their duty had they remained passive. As usual, whenever a lunacy case arises, there is a cry for a reform in the law. We readily admit that the late Act is far from perfect, but the Battersea incident has not shown us that any alteration is needed. If there ever were a case in which a parent was imperatively called upon to inquire into the mental condition of his child, it was this. Had the child given any indication of meditating bodily injury to herself or others, what would have been the responsibility of a father who, knowing this, had made no attempt to interfere? But when, in the opinion of the vast majority who can still exercise common sense on the question of the relations of the sexes, the injury is more far-reaching, and entailing, possibly, misery and disgrace upon the child and her relatives, we are told that the parent must sit still and look on. Mr. Lanchester called in the assistance of a gentleman whose character for probity and professional acumen stands among the first in the country. The expert, after hearing the young lady's history and having an interview with her, formed the opinion that she was of unsound mind. No one will deny that it was a case in which, from a parents point of view, as grave results as suicide might follow if there were any delay. Believing, as they did, that the young lady was insane, and that irremediable harm might ensue if she were left unguarded, they at once put her under control by means of an urgency order. Had it been the sister or daughter of any one of us, would we have acted otherwise? The Commissioners were appealed to, and, after seeing the young lady, they ordered her discharge. She is now free to carry out her original intention. The parents believe she is not responsible, and their professional advisers agree with them. The Commissioners decide it against them, and there, at present, the matter rests, but we hope that Miss Lanchester will at least follow the procedure in the Blackwell case. In the meanwhile we offer the parents our deepest sympathy.

